# Endoscopic treatment of vesicoureteral reflux using calcium hydroxyl apatite in dogs

**DOI:** 10.1186/1756-0500-4-14

**Published:** 2011-01-22

**Authors:** Jalal Bakhtiari, Abdol Mohammad Kajbafzadeh, Mahdi Marjani, Abbas Veshkini, Azin Tavakoli, Mohammad Javad Gharagozlou, Amir Niasari-Naslaji

**Affiliations:** 1 Department of Clinical Sciences, Faculty of Veterinary Medicine, University of Tehran, Tehran, Iran; 2 Pediatric Urology Research Center, Department of Pediatric Urology, Children's Hospital Medical Center, Tehran University of Medical Sciences, Tehran, Iran; 3 Department of Clinical Sciences, Faculty of Veterinary Medicine, Islamic Azad university-Garmsar Branch, Garmsar, Iran; 4 Department of Pathobiology, Faculty of Veterinary Medicine, University of Tehran, Tehran, Iran

## Abstract

**Background:**

Injection of biomaterial to suburetral region, using minimally invasive procedure, has become an interesting topic for urologists to treat vesicoureteral reflux. The objective of this study was to evaluate the feasibility of injecting newly introduced calcium hydroxyl apatite to suburetral region, for treating an experimentally induced vesicoureteral reflux in dogs.

**Findings:**

Bilateral vesicoureteral refluxed (VUR) mixed breed dogs (n = 12; 10-15 kg live weight, 3-6 months of age) were selected for this study. The presence and grade of the reflux were determined using cystography. Accordingly, 6 dogs displayed grade 1 & 2 and the other 6 showed grade 3 & 4 bilateral VUR. Every single dog, with bilateral VUR, underwent endoscopic treatment and received an injection of calcium hydroxyl apatite (an Iranian made product) into the left (treated side) and an injection of the similar volume of normal saline in to the right (control side) subureteric space. One week, 3 and 6 months after treatment, cystography was performed. On each occasion, 4 dogs were euthanized by gas inhalation and biopsy samples were collected for histopathological study from ureter, bladder, kidney, lung and spleen in order to investigate the biomaterial migration into different organs. Data were analyzed using Chi-squared test. In control sides, radiographs confirmed the same grade of VUR, found at the initiation of the study. VUR was resolved in 100% (6/6) of Grade 1 & 2 and 83.33% (5/6) of Grade 3 & 4 in treated side. Therefore, the total success rate of this study was 91.67% (11/12). Macroscopic examination of the vesicouretral region of the treated side revealed a firm and consistent biomaterial mass at the site of injection. Histological findings confirmed inflammation at treated side. In contrast, there was no tissue reaction on control side. There was no evidence for biomaterial migration in macroscopic and microscopic observations in this study.

**Conclusion:**

In the present study, a new biocompatible material produced a firm, consist and sustainable biomaterial mass in the suburetral region for treating vesicouretral reflux without any evidence of biomaterial migration.

## Findings

Application of Biomaterials for treating vesicoureteral reflux is not fully elucidated [1-3]. Primary vesicoureteral reflux is considered as a congenital defect resulting from a short intramural ureteral tunnel and an absence of adequate detrussor behind the intravesical ureter [1,4-7]. Reflux is usually discovered during radiological investigation in 30-50% of children referred with urinary tract infection and the treatment is either medical or surgical [2]. Medical approach may be able to resolve a low-grade reflux, but surgical intervention is required in a complicated reflux with dilated ureter. The minimally invasive procedures, like sub-ureteral injection of biodegradable and biocompatible materials, have become the treatment of choice for reflux in some centers. The success of injecting biomaterials into sub-ureteral region depends on the experience of the surgeon to identify the ureteral orifice [8]. This approach is indicated for patients with poor prophylactic compliance, after failed ureteral reimplantation, in children with naturopathic bladder dysfunction, in those that open surgical techniques are less likely to produce a successful result and in persistent reflux after complicated augmentation or reconstruction procedures [1,9,10]. The rationale behind the endoscopic correction of VUR is to create a solid support behind the intravesical ureter by endoscopic injection of biomaterial, in order to elongate the interamural part of the ureter. This method is simple and can be completed in less than 15 minutes [1,3,5]. The subureteric injection method for reflux was first reported in 1981 [4]. The endoscopic treatment was popularized in the mid-to-late 1980s and became known as the subureteric Teflon injection called STING. Biomaterials used for this purpose, have to be biocompatible, non-toxic and unable to migrate to other organs. Research is concentrated on elucidating the quantity of the biomaterial, the site of injection, local reaction and potential migration to distant organs. Many natural materials have been tested experimentally with advantages and disadvantages [3,11-13]. The objective of this study was to investigate the effect of the newly introduced biomaterial, calcium hydroxyl apatite (an Iranian made product), on treating experimentally induced vesicoureteral reflux, using minimally invasive procedure, in dogs.

The present study was approved by the ethics committee of Faculty of Veterinary Medicine, University of Tehran (BNS714/12.03.07). Healthy dogs (n = 12), weighting 10-15 kg and aged between 3 to 6 months were selected and kept under standard management condition. Rigid cystoscope 13 Fr (3 Fr. Working channel) sheath was used to pass into urethra.

**Figure 1:**
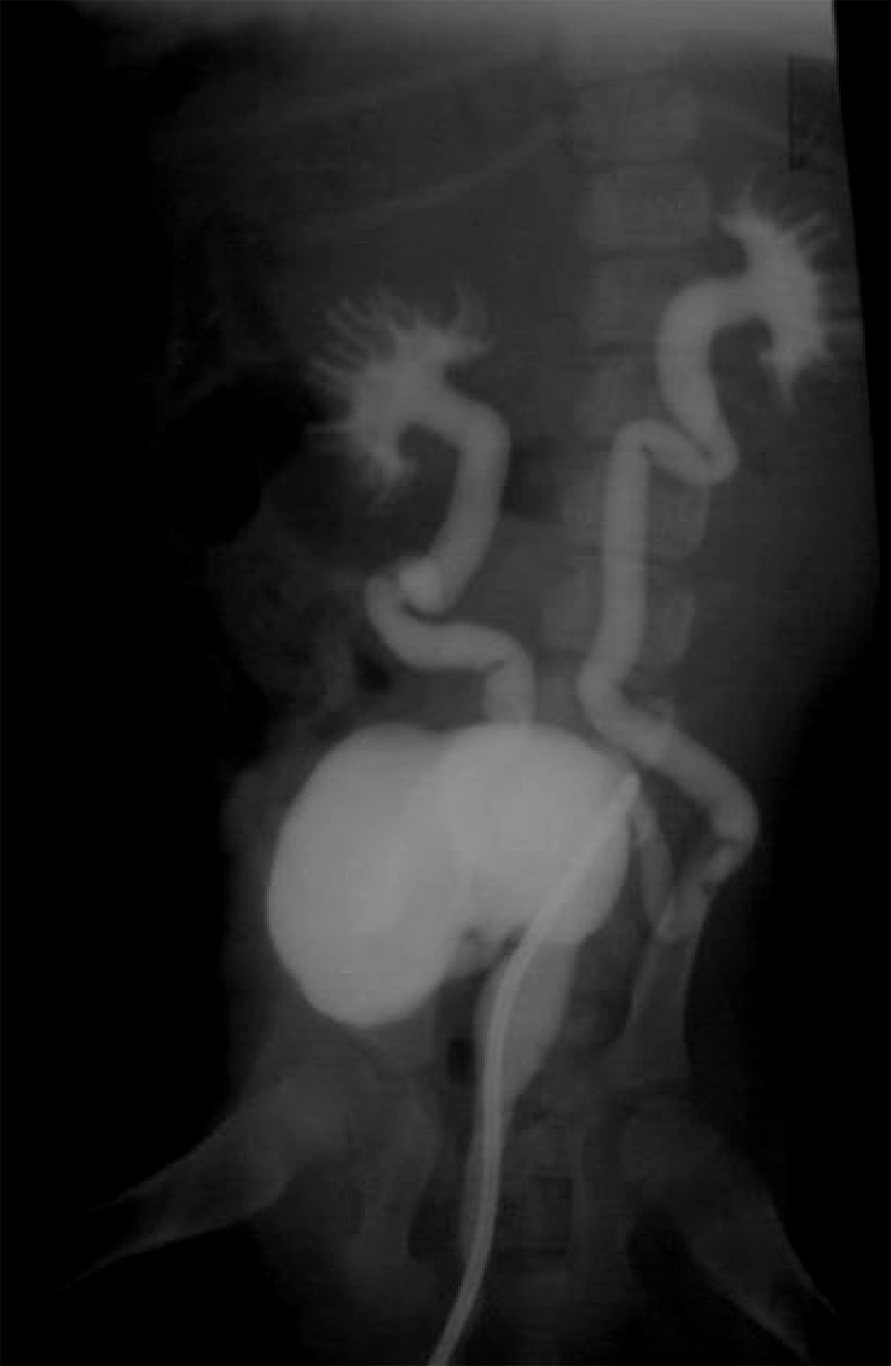
**Cystogram showing induced bilateral reflux**.

In order to induce reflux, the bladder was exposed extraperitoneally through midline incision, and the ureteral orifices were identified and catheterized with 3Fr ureteral stent and reflux was induced by slitting the anterior roof of both intramural ureters for 5-7 mm. The mucosa of ureter and bladder were sutured to each other with 3/0 nylon suture material. Then, the ureteral catheters were removed and bladder was closed with 3/0 absorbable suture material in a routine two layer manner [14,15]. One month after inducing reflux, cystography was carried out under light general anesthesia to confirm the vesicoureteal reflux. Meglumine compound was allowed to run into the bladder until complete distention of bladder achieved. X-ray was taken in lateral and ventrodorsal positions. One month after inducing reflux, endoscopic treatment was performed by calcium hydroxyl apatite (an Iranian made product; Figure 1). The presence and grade of the reflux were determined prior to endoscopy using cystography. Accordingly, dogs were classified to have Grade 1 (n = 3), Grade 2 (n = 3), Grade 3 (n = 3) and Grade 4 (n = 3) bilateral VUR.

**Figure 2:**
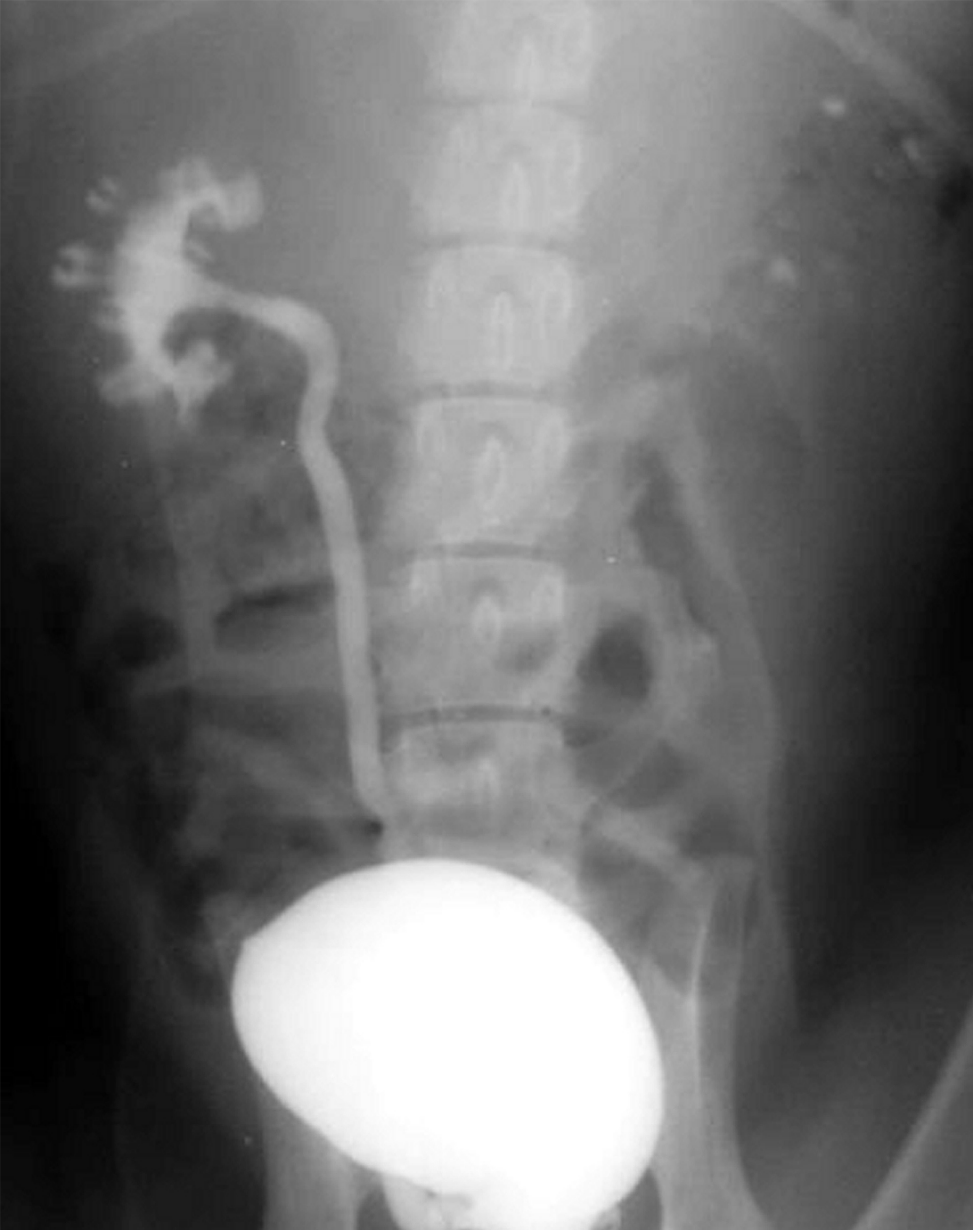
**Cystogram showing endoscopic treated side with calcium hydroxyl apatite**.

Every single dog, with bilateral VUR, underwent endoscopic treatment and received an injection of calcium hydroxyl apatite (an Iranian made product) into the left (treated side) and an injection of the similar volume of normal saline in to the right (control side) subureteric space (Figure 2). The injected volume was 0.4 - 0.6 ml. Mean particle size of calcium hydroxyl apatite was 10.28 (8.31-14.12) um. Once the particles being dissolved and aggregated with autologous serum, it could become as large as 70-120 um. For preparation of this biomaterial the use of glycolic acid, hylorunic acid and any other chemical agents is not necessary.

One week, 3 and 6 months after treatment, cystography was performed. On each occasion, 4 dogs were euthanized by gas inhalation and biopsy samples were collected for histopathological study from ureter, bladder, kidney, lung and spleen in order to investigate the biomaterial migration into different organs. Data were analyzed using Chi-squared test.

In the present study, reflux was successfully induced in all animals. Following inducing reflux, temperature, pulse and respiration rate were in the normal range. However, blood tinged in the urine in the first day and depression and anorexia for few days after treatment were noted which seemed to be expected for such procedures. Dogs return to normal feed few hours after inducing reflux. Endoscopy performed one month after operation, when all dogs were in good health and condition. After conformation of reflux, endoscopic injections of normal saline and calcium hydroxyl apatite into the subureteric space were conducted without any complications such as urine retention or urethral obstruction.

Radiographs obtained one week, 3 and 6 months after treatment confirmed the same grade of VUR in control sides. However, VUR was resolved in 100% (6/6) of Grade 1 & 2 and 83.33% (5/6) of Grade 3 & 4 in treated side. Therefore, the total success rate in this study was 91.67% (11/12) for treated sides compared to 0% (0/12) for control sides (P < 0.01). Macroscopic examination of the vesicouretral region of the treated side revealed a firm and consistent biomaterial mass at the site of injection.

**Figure 3:**
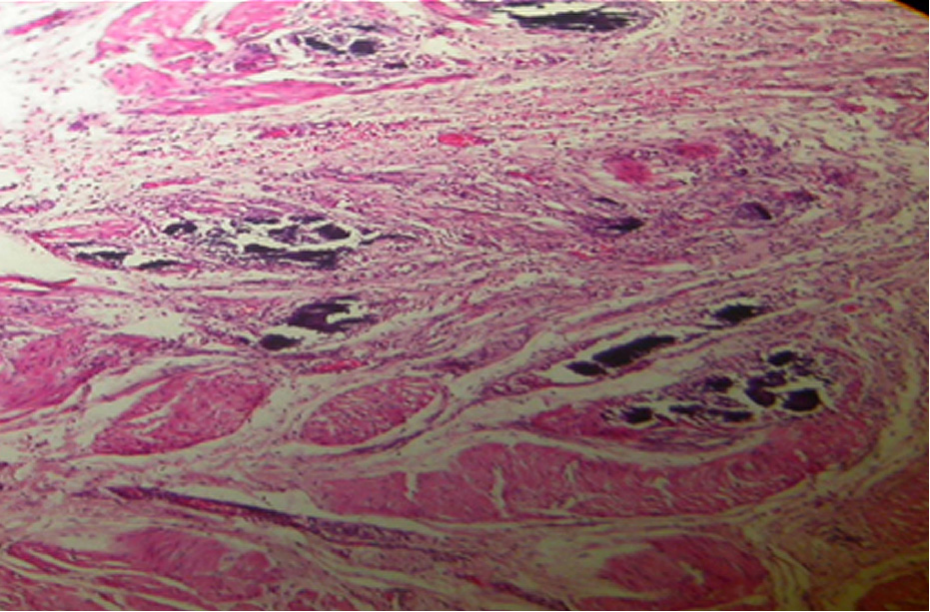
**Bladder wall showing sedimentation of calcium hydroxyl apatite (H & E×100)**.

**Figure 4:**
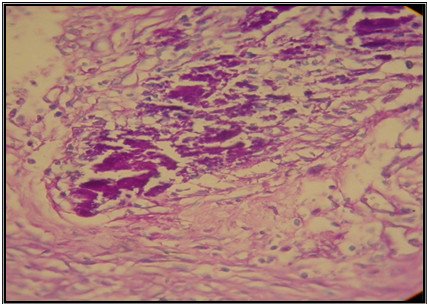
**Small violet granules of calcium hydroxyl apatite in the site of injection (H & E×400)**.

**Figure 5:**
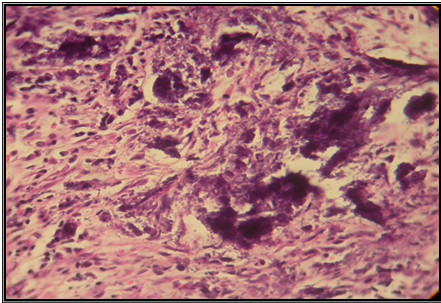
**PAS staining with purple sedimentation of calcium hydroxyl apatite (PAS×100)**.

**Figure 6:**
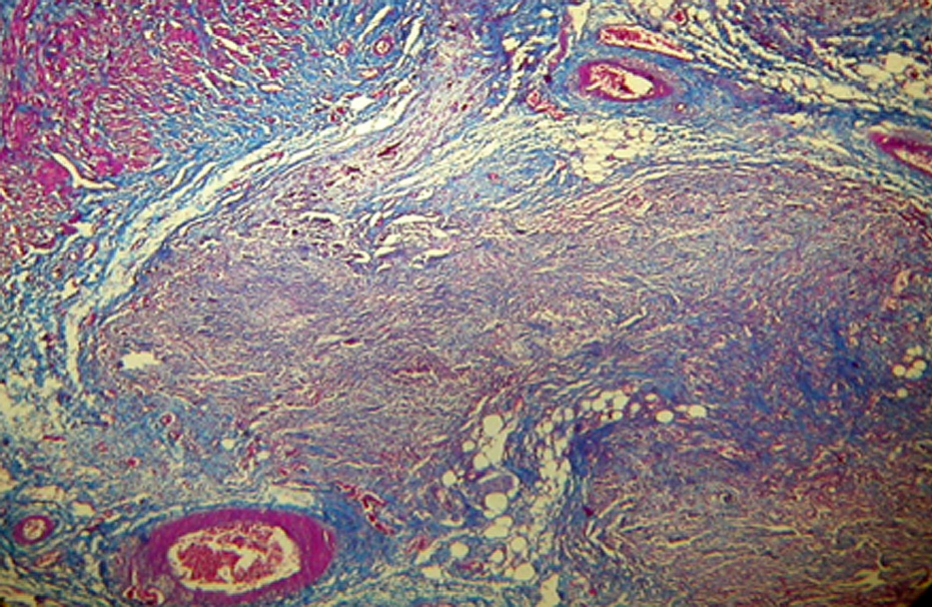
**Trichorom staining of blue line color showing fibrosis in the site of calcium hydroxyl apatite injection (trichorom×40)**.

Histological findings following the first week after treatment revealed inflammation, collagen formation, edema and/or fibrosis of the ureter or bladder wall at treated side (Figure 3, 4, 5 and 6). There was no tissue reaction on control side. There was no evidence for biomaterial migration in macroscopic and microscopic observations in this study.

All urine cultures for the 12 dogs of bilateral reflux were sterile throughout the study. No migration of biomaterial was found in other organ. There was no residue of calcium hydroxyl apatite in different samples obtained from different organs. After 6 months, the gross appearance of injection sites revealed well circumscribed as elevation of the mucosa without any overlying erythema, fibrosis or ulceration. Gross examination of the vesicoureteric region showed a well circumscribed, firm and consistent subureteric calcium hydroxyl apatite mass, retaining its shape and position.

The purpose of this study was to evaluate the safety, efficacy, local tissue reaction and possible migration of new calcium hydroxyl apatite to treat dogs with induced reflux using endoscopic intervention. The procedure of inducing VUR used in this study was the same as the procedure used previously in pigs by slitting the anterior roof intramural ureters followed by suturing [16-18]. The endoscopic correction of VUR has become widely accepted as a standard procedure to many specialists [11]. The main concern in using biomaterials is not the procedure of injection. In the present study, injection of the biomaterial was simple and fast procedure which was comparable to the result of other investigators [5,11,12,19]. But the main concern is the quality and safety of biomaterials and to avoid the potential danger of injecting foreign materials [20]. Many materials such as blood, fat and cartilage have been tested experimentally [1,3,4]. One of the main concern in using biomaterials is the migrations of these materials to other organs, like muscle layer and/or submucosal layer of urinary bladder, spleen and lung which have been reported following endoscopic treatment of urethral reflux in dogs, rabbits and human [3,18,21-23]. Therefore, long term follow up seems necessary to approve the efficacy of different biocompatible materials used in VUR treatments. In the present study, after 6 months, there was no evidence of migration of calcium hydroxyl apatite from the injected site to other organs. Similar result, but not for long term, was found using iodine labeled Dextranomer based implant [22]. The volume of injected material was 0.4-0.6 ml in the present study with high success rate (91.67%). In other studies, less volume of biomaterials (0.1-0.4 ml) was used [11,12,21] but the success rate was lower than the present study (53%; 11).

## Conclusion

An Iranian made calcium hydroxyl apatite, which is extremely less expensive than commercially available biomaterials, was efficient to correct reflux without any evidence of complications. Further investigation is required to evaluate this biomaterial for human.

## Competing interests

The authors declare that they have no competing interests.

## Authors' contributions

JB supervisor of this project performing experimental induction of reflux. AMK carried out endoscopic treatment of reflux and helped in design of the study. MM residential student to this project collecting data's and participated in the sequence alignment. AV cystouretrogram interpretation. AT carried out drafting of the manuscript and participated in the sequence alignment. All authors read and approved the final manuscript.

The authors declare that they have no competing interests.
